# Use of Vending Machines to Deliver Oral Rapid HIV Self-Tests to Veterans: Protocol for a Pilot Study

**DOI:** 10.2196/84317

**Published:** 2026-01-29

**Authors:** Tessa Rife-Pennington, Michael P Douglas, Wendy Xie, Jennifer Cocohoba

**Affiliations:** 1 Pharmacy Service, San Francisco VA Health Care System, 4150 Clement St (119), San Francisco, CA, 94121, United States, 1 415-319-1193; 2 Department of Clinical Pharmacy, School of Pharmacy, University of California, San Francisco, San Francisco, CA, United States; 3 Department of Pharmacy Practice, Thomas J Long School of Pharmacy, University of the Pacific, San Francisco, CA, United States

**Keywords:** veterans, HIV, self-testing, sexually transmitted diseases, vending machine

## Abstract

**Background:**

California has the largest number of people living with HIV in the United States, and in 2022, there were 4882 new diagnoses. Veterans with histories of substance use, viral hepatitis, sexually transmitted infections, and homelessness carry substantial HIV burden. Testing is essential, yet approximately 12% of Californians with HIV were undiagnosed in 2020, and 50% of veterans in care had never been tested as of 2023. HIV self-tests (HIVSTs) can mitigate stigma, confidentiality, and access barriers, and vending machines (VMs) offer private, convenient distribution. However, the use of VM-dispensed HIVST has not been evaluated for veterans or within Veterans Affairs (VA) settings.

**Objective:**

We describe a Reach, Evaluation, Adoption, Implementation, and Maintenance–guided pre-implementation protocol to evaluate VM-dispensed HIVSTs in Northern California VA clinics and supportive housing settings.

**Methods:**

Fifteen VMs will stock oral-fluid HIVSTs (n=900). Program data (de-identified dispense logs), veteran electronic surveys (n=90), and qualitative interviews (n=15) will quantify reach (uptake), early effectiveness proxies (use, results, and next steps), adoption (machine/site dispensing), implementation (stockouts, restocking interval, and costs), and maintenance (dispensing trends).

**Results:**

Ethics approval activities (study material development and Institutional Review Board submission) began 2 months prior to the receipt of award funding (January to February 2025). Following funding, the project is planned for over a total of 18 months (12 months original project period + 6 months no-cost extension; March 2025 to August 2026). Ethics approval was obtained in August 2025. Veteran feedback was incorporated into study materials, and HIVSTs were purchased and packaged in September to November 2025. HIVSTs were added to VMs, and data collection is projected to occur from December 2025 through June 2026. Results are anticipated to be available in August 2026.

**Conclusions:**

This study will generate practice-ready evidence on the feasibility, acceptability, and early behavioral impacts of VM-dispensed HIVSTs for veterans. By pairing a stigma-responsive delivery channel with pragmatic measures, findings can inform equitable scale-up across VA and community settings, guide comparative evaluations of distribution channels (VMs, mail-to-home, or clinic pick-up), and support privacy-preserving linkage strategies to confirmatory testing, HIV pre-exposure prophylaxis, and treatment. Results will address a critical evidence gap for veteran-focused HIV prevention and provide parameters for multi-site evaluations.

## Introduction

San Francisco and Alameda Counties, core jurisdictions in the United States Ending the HIV Epidemic initiative, continue to experience concentrated HIV burden among populations served by the San Francisco Veterans Affairs Health Care System (SFVAHCS) [1]. Despite national recommendations for routine and risk-based screening, testing within Veterans Affairs (VA) care centers remains suboptimal, with many veterans never having been tested [2,3]. Addressing this persistent screening gap requires approaches that extend beyond traditional clinic workflows and that better reflect how veterans prefer to seek prevention services.

Multiple barriers impede routine testing in VA and community settings alike. Veterans report concerns about confidentiality and stigma, competing demands during clinical visits, uncertainty about eligibility or need for testing, and logistical hurdles such as travel and wait times [4]. Clinicians cite limited time, competing priorities, and variable comfort initiating testing discussions [5]. Framed through a behavioral lens, these barriers cluster around privacy, autonomy, convenience, and perceived control—constructs linked to testing avoidance and delayed diagnosis.

HIV self-tests (HIVSTs) are a recommended complement to facility-based testing and can mitigate several of these barriers by enabling private, user-directed testing [1,6-11]. Distributing HIVSTs through vending machines (VMs) further increases anonymity and convenience, while leveraging existing low-touch infrastructure. Early experience from non-VA settings suggests VM distribution is feasible and acceptable; however, its utility for veterans, and its implementation within VA facilities and partnered housing sites, have not been evaluated [12-16].

This protocol responds to this gap by describing a mixed-methods evaluation of VM-dispensed HIVSTs within the SFVAHCS and affiliated supportive housing locations. Guided by the Reach, Evaluation, Adoption, Implementation, and Maintenance (RE-AIM) framework, this study will characterize reach, adoption, and implementation at the site level and assess early individual-level behaviors related to test use [17]. By pairing a stigma-responsive delivery channel with pragmatic measures, this work aims to generate actionable evidence for VA and community partners considering VM-enabled HIV screening.

## Methods

### Study Aims and Objectives

This is a single-arm, mixed-methods pilot implementation evaluation that aims to evaluate the RE-AIM of VM-dispensed HIVSTs among Veterans located in Northern California (Table 1) [17].

**Table 1.** Study aims and hypotheses.

Aim	Description	Hypothesis
Aim 1: Reach	Characterize HIV risk factors among veterans who obtained ≥1 VM^a^-dispensed HIVST^b^.	Among survey respondents, commonly reported HIV risk indicators will include male-to-male sexual contact, condomless sex, and unregulated drug use in the past 6 months.
Aim 2: Effectiveness (early outcomes; not causal)	Describe early, self-reported outcomes after obtaining a VM-dispensed HIVST (eg, intended use, completed use, results, and planned next steps) and the perceived usefulness of embedded HIV resources.	Most participants will report intending to use or having used the HIVST, will report reviewing the included post-test resources, and will report an intention to seek confirmatory testing and/or prevention services (eg, HIV pre-exposure prophylaxis).
Aim 3: Adoption	Identify which VM locations have the highest HIVST dispensing volume and when dispensing occurs (eg, clinic vs supportive housing; weekday vs after hours).	HIVST dispensing will be the highest at locations with higher baseline VM utilization and/or higher concentration of veterans experiencing housing instability (eg, supportive housing sites or outpatient clinics serving veterans experiencing homelessness).
Aim 4: Implementation and Maintenance	Assess implementation outcomes (eg, direct costs) and acceptability/experience of veterans with VM-dispensed HIVST (eg, convenience, privacy, satisfaction, and preference compared with other access points).	Participants will rate the VM delivery method as highly acceptable (eg, convenient, private, and satisfactory) and will describe VM access as a low-barrier, low-stigma option for obtaining HIV testing; dispensing will be feasible to maintain over the pilot period with measurable restocking workflows.

a VM: vending machine.

b HIVST: HIV self-test.

To accomplish this, we will collect veteran self-report data from 15 VMs via an electronic web-based Questionnaire 1 (n=90), a semi-structured qualitative interview paired with Questionnaire 2 (n=15), and HIVST distribution data (n=900) via the EMS software (VendNovation LLC). Our study lacks a comparison condition. Accordingly, the “effectiveness” outcomes are limited to early, self-reported behaviors and perceived usefulness/acceptability; causal inference regarding changes in HIV testing, diagnosis, or linkage to care is not possible.

### Timeline

The study is expected to be completed over 20 months (reflecting 2 months of pre-funding activities, 12 months for the original project period, and a requested 6-month no-cost extension). During months 1‐2 (January to February 2025), research team members designed initial study materials and submitted Institutional Review Board (IRB) protocols for review. During months 3‐8 (March to August 2025), requested edits to IRB protocols were incorporated, and IRB approval was attained. During months 9‐11 (September to November 2025), veteran feedback was obtained about the study materials, which were further refined and finalized; HIVSTs and VM coils were purchased; and HIVSTs were packaged with the study materials. HIVSTs were added to the VMs in December 2025. Data collection is projected to occur in months 12‐18 (December 2025 to June 2026), and data analysis is anticipated to be performed in months 19‐20 (July to August 2026).

### Setting

This study will be conducted at the SFVAHCS, which serves over 310,000 US Veterans at the San Francisco VA Medical Center and 9 community-based outpatient clinics located in Downtown San Francisco, Oakland, San Bruno, Santa Rosa, Clearlake, Ukiah, and Eureka, California. HIVSTs will be stocked in pre-existing SFVAHCS VMs: 2 are located at the San Francisco VA Medical Center, 7 in community-based outpatient clinics (all the cities listed above except San Bruno), and 6 in supportive housing buildings where veterans live (San Francisco and Daly City, California). Research study team members will conduct qualitative interviews with veterans at the San Francisco VA Medical Center and supportive housing buildings. The research study team will also be available via telephone and video to assist with recruitment, enrollment, and collection of study measures.

### Participant Eligibility and Recruitment

To be eligible for Questionnaire 1, participants must be a US Veteran (of any service discharge status), must be registered for VM access, and must have taken a HIVST from one of the 15 SFVAHCS VMs. To be eligible for the qualitative interview and Questionnaire 2, veterans must meet the eligibility criteria for Questionnaire 1 and must have personally used a VM-dispensed HIVST. Non-veterans who obtain a VM-dispensed HIVST from a veteran will be ineligible. Recruitment fliers will be taped on the VMs, HIVSTs, and posted in common areas of supportive housing buildings. The research team will attend veteran community meetings at supportive housing buildings to introduce the study and recruit participants.

### Ethical Considerations

We obtained approval by the University of California IRB and the San Francisco Veterans Affairs Human Subjects Committee in August 2025 (No. 25-43931). The research study team will obtain electronic or written informed consent before beginning the study questionnaire and qualitative interview. Participants will receive a US $20 electronic gift card after completion of Questionnaire 1 (maximum of US $40 per participant). Veterans who completed Questionnaire 2 would receive a US $100 gift card. Each veteran advocate will receive a US $1000 honorarium for their time and expertise (about 5 hours of work). HIVST dispensing data will be de-identified and tracked regularly throughout the study using HRVM software. All participant data collected will be de-identified to ensure privacy and confidentiality.

### Study Measures and Outcomes

Study measures and outcomes will be mapped onto the RE-AIM framework for evaluating individual and organizational factors that determine public health impacts of a program or policy (Table 2) [17].

**Table 2.** Application of the RE-AIM^a^ framework to the proposed study.

Dimensions	Measures/Outcomes	Data source	Data level
Reach the target population	Number of participants who access ≥1 HIVST^b^	HRVM software (EMS, VendNovation LLC)	Organizational
	Participant demographics and characteristics, sexual partners and practices, drug use practices	Electronic Questionnaires 1 and 2	Individual
Effectiveness or efficacy	Participant HIVST usage plan, results, and steps after testing	Electronic Questionnaire 1	Individual
Adoption by target staff, settings, systems, and communities	Number of HIVSTs dispensed, location, date, and time	HRVM software (EMS, VendNovation LLC)	Organizational
Implementation consistency, costs, and adaptations made during delivery	Direct costs (vending machine coils and HIVSTs)	Purchase order invoices	Organizational
Maintenance/sustainment of intervention effects in individuals and settings over time	Participant reported outcome measures	Electronic Questionnaire 1; semi-structured qualitative interview	Individual

a RE-AIM: Reach, Evaluation, Adoption, Implementation, and Maintenance.

b HIVST: HIV self-test.

The RE-AIM framework has been utilized in similar studies evaluating health care provider views on HIVSTs, workplace HIV testing, integrating HIV prevention services into family planning, barriers and facilitators to HIV pre-exposure prophylaxis uptake, and provision of conditional financial incentives to people living with HIV [18-22].

### Study Instruments

Questionnaires 1 and 2 will be developed in Qualtrics in January 2026, and both will evaluate self-reported sociodemographic characteristics, including age; gender identity; race/ethnicity; sexual orientation; branch of military service; service-connected disability rating; past 6-month housing status, sexual practices, and street drug use; and if ever heard of, willing to take, or has taken HIV pre-exposure prophylaxis, HIV post-exposure prophylaxis, or doxycycline PEP in the past year. These questions will be adapted from the Collecting Demographic Data at Syringe Services Programs guide, Core Questionnaire, internal VA guidance on taking a brief sexual health history, and self-efficacy scale for HIV risk behaviors [23-25]. Questionnaire 1 will include additional questions, including HIVST usage plan, results, and next steps. Added questions will be derived from the Theoretical Framework of Acceptability Questionnaire, which evaluates affective attitude, burden, ethicality, intervention coherence, opportunity costs, perceived effectiveness, and self-efficacy of health care interventions, as well as one item from the Net Promotor Score “How likely is it that you would recommend to a friend or a colleague?”; they will be asked to rate convenience, privacy, satisfaction, reduced stigma, preference over other testing methods, ability to pay for testing, and other recommended self-tests to add to the VMs (eg, chlamydia/gonorrhea self-swabs) [26,27].

A semi-structured qualitative interview guide will be developed. Participants will be asked about prior HIV testing experiences, knowledge of self-testing, experience accessing via VMs, perceived ease of use, motivations for self-testing, concerns, impact of stigma, support needs, and recommendations for other self-test distribution via VMs [28].

### Veteran Advocates

We will use participatory action research methodology and recruit 4 veteran advocates with lived/living expertise of HIV who reside in California to provide feedback and recommendations on study materials and assist with interpretation and dissemination of study findings [29-31]. Veterans will provide feedback and recommendations on study recruitment materials, education handouts, and instruments (Questionnaires 1 and 2 and the qualitative interview guide) via a feedback form and a virtual focus group (Multimedia Appendix 1). Feedback will be incorporated prior to resource utilization. Each veteran advocate will receive a US $1000 honorarium for their time and expertise (about 5 hours of work).

### Study Administration and Participant Reimbursement

Veterans who dispensed a HIVST via VMs may anonymously complete Questionnaire 1 via web-based link located on the HIVST. Participants will receive a US $20 electronic gift card after completion (maximum of US $40 per participant). Reimbursement will be available for a total of 90 completed questionnaires.

Veterans (n=15) will meet with the research team members for approximately 1 hour to complete an in-person semi-structured qualitative interview. Veterans will need to complete Questionnaire 2 via an iPad, and those who did so would receive a US $100 gift card. Veterans may complete Questionnaire 1 and/or the semi-structured qualitative interview visit (both are not required).

### Data Collection and Storage

Interviews will be audio recorded via Microsoft Teams, validated by research team members, and submitted for transcription [32]. Collected Qualtrics questionnaire and semi-structured qualitative interview data will be secured in password-protected files on the SFVAHCS network in January 2026, and access will be restricted to research study staff. Participants will be assigned alpha-numeric identifiers, and files with identifying information will be destroyed upon completion. HIVST dispensing data will be de-identified and tracked regularly throughout the study using HRVM software (EMS, VendNovation LLC).

### Sample Size and Precision

This pilot implementation evaluation is designed to estimate feasibility and acceptability outcomes rather than to test efficacy. Funding was allocated for the purchase of 900 HIVSTs. Assuming 100% distribution during the data collection period, we estimated a ~10% optional survey response rate based on a similar HIVST distribution study and targeted up to 90 completed surveys [16]. With 90 survey responses, descriptive estimates of key feasibility/acceptability proportions (eg, intended/actual test use, satisfaction, and perceived privacy) will have acceptable precision (eg, for proportions near 50%, an approximate 95% CI half-width of ~10 percentage points). We will conduct up to 15 qualitative interviews, which is expected to be sufficient to achieve thematic saturation for a focused inquiry on acceptability, barriers/facilitators, and implementation experiences.

### Data Analysis

Descriptive statistics will be used to evaluate participant characteristics and organizational-level data. For the semi-structured qualitative interviews, an inductive coding process will be used employing thematic analysis [33]. Research team members will independently code three veteran transcripts, resolve discrepancies, and refine the initial code book collaboratively. Once the final codebook is generated, transcripts will be divided evenly among team members for coding using qualitative research software (Dedoose, Sociocultural Research Consultants LLC, Version 9.2.22). Coding will be verified via a 10% cross-check to ensure consistency across team members. Patterns in coding, categories, and themes/concepts will be examined to draw conclusions about the data [33].

## Results

Ethics approval was obtained in August 2025. Veteran written (n=4) and focus group (n=3) feedback was obtained in September 2025, and study materials were refined by November 2025. HIVSTs and VM coils were purchased, HIVSTs were packaged with study materials, and all VMs were anticipated to be stocked with HIVSTs by December 2025. Data collection from VM dispensing and semi-structured qualitative interviews are projected to occur December 2025 through June 2026, with final analyses in July to August 2026.

## Discussion

### Principal Findings and Comparison With Previous Works

This protocol addresses a persistent gap in HIV screening among veterans despite US and national VA recommendations for routine or risk-based testing [2,3,34]. With California’s high-burden context, only half of the veterans in care have ever been tested, underscoring the need for alternative, low-barrier testing strategies that can extend reach beyond clinic workflows [35-37]. While HIVSTs are accurate and acceptable across diverse settings, distribution via VMs has not been evaluated in veteran populations, representing a vital evidence gap this study will begin to fill [8-11].

VM-dispensed HIVSTs are a promising, technology-enabled approach precisely because they target known behavioral and structural barriers to testing, including stigma, confidentiality concerns, and competing clinical priorities. By enabling private, on-demand access outside traditional encounters, VM delivery may increase autonomy, convenience, and reduce disclosure concerns, mechanisms frequently identified as drivers of testing avoidance and delayed diagnosis. Empiric work in non-VA settings indicates that VM access to HIVSTs is feasible, acceptable, and capable of engaging first-time testers across public venues and community spaces; adapting this model to VA facilities has potential to extend reach while ensuring privacy and choice [12-16].

Our mixed-methods design, mapped onto the RE-AIM framework, is intended to generate implementation-ready knowledge [17]. Quantitatively, dispensing data from 15 VMs and participant surveys will estimate reach (who accesses tests), early effectiveness proxies (intended/actual use and next steps), adoption (dispensing volume, time, and location), and implementation (direct costs, fidelity, and adaptations). Qualitative interviews will illuminate context, equity, and mechanisms, including how privacy, convenience, and autonomy shape decisions to test and seek confirmatory services, providing critical explanatory depth that can guide scale-up interventions.

Thus, this protocol advances a low-barrier, stigma-responsive strategy to expand HIV screening among veterans by pairing the privacy and convenience of self-testing with the scalability of VM infrastructure. Findings on reach, acceptability, adoption, and early post-test behaviors will provide actionable guidance for VA and community partners considering VM-enabled HIV prevention programs, while delineating the analytic and operational groundwork for larger, multi-site trials.

### Limitations

This study protocol has several limitations. First, participation requires proximity and access to SFVAHCS VMs, which may preferentially include veterans who are already engaged with VA services or comfortable with public VM use. As such, reach estimates may under-represent veterans who avoid clinic spaces or public machines. Second, the evaluation is limited to a single VA health system with clinics and supportive housing partners in Northern California; differences in housing policies, facility layouts, and community norms may constrain adoption and transferability to other VA and non-VA settings. Third, private self-testing and reliance on self-reported survey/interview data introduce recall bias, social desirability bias, and limit the verification of reactive results or care linkage. Fourth, inequities in technology access, health literacy, or trust in research staff may shape who participates, potentially biasing acceptability and feasibility estimates. Finally, as a 12-month pilot study, outcomes will emphasize uptake, acceptability, and implementation (RE-AIM) of HIVST distribution via VMs, rather than characterize HIV incidence, engagement in care, or sustained outcomes.

### Future Directions

Findings from this pilot should inform a multi-site VA evaluation that tests scale-up strategies and the comparative effectiveness of distribution channels (VMs vs mail-to-home vs clinic pick-up) on reach, equity, and care linkage. A pragmatic stepped-wedge or cluster randomized design across VA and supportive housing partners would allow the estimation of system-level effects while accommodating staged implementation. Future work should also examine reductions in stigma, increased autonomy/convenience, and changes in self-testing efficacy among veteran subgroups (eg, women and gender-diverse veterans, those experiencing homelessness, rural vs urban settings, and diverse racial/ethnic groups).

To strengthen downstream outcomes, next-phase studies could embed privacy-preserving navigation options (eg, quick response [QR] codes or links inside HIVSTs that directly connect veterans to follow-up testing and HIV pre-exposure prophylaxis). Future economic evaluations (implementation costs, and budgetary impact) will be vital for sustainability decisions. Implementation research should compare site-level strategies (staff champions, restocking workflows, and accessibility features) and assess maintenance over time (supply chain reliability, vandalism, theft, policy, and contracting steps). Finally, bundling HIVSTs with sexual health resources (pregnancy tests or syphilis self-tests) warrants further testing to evaluate whether integrated harm-reduction VMs increase first-time HIV testing and accelerate linkage to preventive care and treatment.

## Supplementary material

10.2196/84317 Multimedia Appendix 1 A focus group guide that will be used to interview 4 veteran advocates. The guide includes an overview of the focus group (participants, materials, roles and responsibilities), bulleted outline, facilitator script, references, confirmation of notice of participation, recruitment tracking form, and reviewer feedback form.

10.2196/84317 Peer Review Report 1 Peer Review report from California HIV/AIDS Research Program (CHRP) Review Committee.

## Abbreviations

HIVST: HIV self-test
IRB: Institutional Review Board
QR: quick response
RE-AIM: Reach, Evaluation, Adoption, Implementation, and Maintenance
SFVAHCS: San Francisco VA Health Care System
VA: Veterans Affairs
VM: vending machine
