# Supplementary material for: Use of Vending Machines to Deliver Oral Rapid HIV Self-Tests to Veterans: Protocol for a Pilot Study
Source: JMIR Res Protoc. 2026 Jan 29;15:e84317. doi: 10.2196/84317 (PMC12855721; doi:10.2196/84317)
Supplement: Multimedia Appendix 1 [file resprot-v15-e84317-s001.pdf]

Focus Group Guide

Veteran participants:

- Inclusion Criteria: United States (US) Veteran, any healthcare eligibility status, with a diagnosis of HIV, currently living in California
- Exclusion Criteria: Individuals who are not a US Veteran, who do not have a diagnosis of HIV, or who live in states other than California
- Recruitment:
  - San Francisco VA Health Care System (SFVAHCS) clinicians involved in treatment of Veterans living with HIV may nominate key individuals they think would make good focus group participants. SFVAHCS clinicians will contact Veterans to obtain verbal approval for the study team to contact them regarding participation.
  - Study team will contact Veterans by phone/email to confirm interest, email/mail interested Veterans a confirmation notice with instructions (**Appendix A**) and materials, assign a participant number, and document in **Appendix B**.
- Total members: 4
- Time required: 4 hours of prep, 1 hour for the focus group, option to participate in written products (conference abstracts and presentations, manuscripts)
- Payment: $1000 honorarium (check mailed)

Materials:

- Feedback form (**Appendix C**)
- Recruitment fliers
- Education handouts
  - HIV 101
  - Rapid Oral HIV Test Fact Sheet
  - TakeMeHome Handout
  - HIV testing and treatment resources by county: Alameda, Humboldt, Lake and Mendocino, San Francisco, and Sonoma Counties
- Qualtrics survey
- Qualitative interview guide
- W9 form for honorarium

Roles and responsibilities:

- Moderator:
  - Welcome participants as they arrive
  - Create a warm and friendly environment, use purposeful small talk
  - Provides the introduction
  - Sets ground rules
  - Asks questions with pauses and probes
    - 5 second pause
    - Probes:
      - "Could you explain further?"
      - "Could you give an example?"

            - "Help me understand what you mean."
        - Restates/summarizes participant responses and clarifies to ensure understanding
    - Listens attentively with empathy
    - Keeps personal views/bias out of the facilitation
    - Uses subtle group control and expertly manages group challenging dynamics
        - Experts: "Thank you. What do other people think?"
        - Dominant talkers: "Let's have some other comments."
        - Shy participants: "What do you think about this topic?"
        - Quiet participants: Ask to repeat response more loudly
        - Ramblers: Interject when they inhale/briefly pause
    - Three-step conclusion
        - Summarize discussion
        - Review purpose for meeting, ask if anything was missed during the discussion
        - Thanks and ending of meeting

- <u>Assistant moderator/mentee</u>:
    - Change all Veteran names in Zoom to First name and last initial
    - Take notes during the discussion
        - Listed for notable quotes, well-said statements that illustrate an important point of view
        - Document key points/themes for each question
        - Identify relevant follow-up questions to ask that the moderator may have missed
        - Track helpful insights/concepts (e.g., lightbulb moment)
        - Make note of factors that may help with analysis (e.g., passionate comments, clues that indicate a level of agreement, support, or interest)
    - Show cc (captions) in the Zoom meeting
    - Monitor the text chat
    - Ask questions and contribute when invited
    - Debrief with moderator afterwards

- <u>Veteran participants</u>:
    - Prior to the focus group session:
        - Review the recruitment fliers.
        - Complete the Qualtrics survey.
        - Review the qualitative interview guide.
        - Take notes on the feedback form and email to team.
        - Complete and email W9 form.
    - Attend the focus group discussion.
        - Be prepared to discuss feedback from with the group.

Bulleted Outline

Welcome

- Introduce moderator and mentee

Purpose

- We are conducting a study…
- The results will be used for…
- You were selected because…

Ground rules

- Tape recording
- We want to hear from all of you
- There are no right or wrong answers
- What is said in this group stays here
- Participation and attention
- Check in

Questions

- Recruitment fliers
- Educational handouts
- Qualtrics survey
- Interview guide

Wrap-up

Script

Welcome

- "Thank you for agreeing to be a part of this focus group. We truly appreciate your willingness to participate."

Introductions

- "My name is [insert name]. I am a [insert title/credentials]. I will be moderating today's session. I'm joined today by [insert name, title/credentials. [insert name] is our assistant moderator for today's group."

Purpose

- "We are conducting a paid research study at UCSF and San Francisco VA to learn about Veteran interests, experiences, and perspectives accessing and using HIV self-tests from 15 harm reduction vending machines. These vending machines are located at the San Francisco VA Medical Center, in 7 community-based VA outpatient clinics, and in 6 supportive housing buildings where Veterans live.
- We will attach each HIV self-test with a packet of educational handouts. These focus on what is HIV, how to prevent HIV, using HIV self-tests, and where to access further HIV testing and treatment.
- Veterans who access a HIV self-test from a vending machine will have the opportunity to take an anonymous online survey about their experience and receive $20.
- In addition, we are interviewing 15 Veterans who access and use a HIV self-test, and they will receive $100.
- The purpose of this focus group is for you to provide feedback on the study recruitment fliers, online survey, interview script, and the education handouts. We will use what we learn to make vital updates and inform study procedures. You may also have the opportunity to contribute to written products that come from this research, such as conference abstracts, posters, and presentations, as well as written manuscripts.
- We value your time and expertise. We want you to share your honest feedback and opinions with us."

Ground rules

- *Tape recording*
    - "We want to capture everything you have to say. We will be tape recording this discussion with videos off. The recording will be saved as a written transcription.
    - We assigned each of you a participant number. [insert name] will be updating your name in Zoom to reflect your number. That way our written transcription remains anonymous.
    - We will not identify anyone by name in our analysis."
- *We want to hear from all of you*
    - "We would like for everyone to participate.
    - I may call on you if I haven't heard from you in a while.
    - Make sure that only one person speaks at a time so that everyone has a chance to contribute."

- *There are no right or wrong answers*
    - "All person's experiences and opinions are important.
    - You do not have to agree with each other, but you must listen respectfully as others share their views.
    - Speak up to whether you agree or disagree.
    - We want to hear a wide range of opinions."
- *What is said in this group stays here*
    - "We want you to feel comfortable sharing when sensitive issues come up.
- *Participation and attention*
    - "We ask that you turn of your cell phones during the discussion. If you must take a call, please do so as quietly as possible and rejoin as quickly as you can."
- *Check in*
    - "Are there any questions before we begin?"

Questions

- <u>Recruitment fliers</u>:
    - "This first discussion will focus on the research study recruitment fliers. The fliers will be attached to each HIV self-test inside the vending machines. There are 7 versions of the same flier, each with a different picture on the upper left.
    - *Content*
        - "Do the fliers answer these key questions?
            - What is the study about?
            - What do you want from me?
            - Why should I care?
            - Who is eligible to participate?
            - Where/how do I get involved?"
    - *Relevance*
        - "Are the images/pictures on the fliers relevant?
        - Are the images/pictures on the fliers representative of:
            - Diverse populations
            - Veterans
            - People living with HIV"
    - *Visuals*
        - "Are the fliers eye catching? Do they grab your attention?
        - Do the fliers have a good balance of images and white space?
        - Do you like the colors and fonts used on the fliers?"
    - *Clarity and structure*
        - "Are the fliers easy to read?
        - Is the content on the fliers easy to understand?
        - Is the language on the fliers too technical?"
    - *Ease of use*
        - "Are the fliers easy to act on?
        - Is having the QR code useful?
            - Is the website useful?
            - Is there another/better way to share information about the study?"
    - *Suggestions for improvement*

        - "What are some things we could do to improve the fliers?
        - How could we make the fliers more engaging?
    - That concludes my questions about the recruitment fliers. Does anyone have any additional feedback they would like to share?"

- <u>Educational handouts</u>
    - "This next section focuses on the education handouts and resources. All education handouts will be packaged with each HIV self-test. The HIV testing and treatment resources will be custom added depending on the vending machine location. For example, resources located in San Francisco County will be added to vending machines located in San Francisco."
    - *General experience and perceptions*
        - "What were your overall impressions of the handouts?
        - What aspects of the handouts did you find more or less relevant?"
    - *Clarity and structure*
        - "Were the handouts clear and easy to understand?
        - Were there any handouts that were confusing or unclear?
    - *Content and relevance*
        - "Were the handouts relevant?
        - Was the content in the handouts an appropriate level? Do we need to provide more educational information?
        - What handouts, if any, would you suggest we remove?
        - What types of handouts should we add?"
    - *Suggestions for improvement*
        - "What are some things we could do to improve the handouts?
        - How could we make the handouts more engaging?
    - "That concludes my questions about the handouts. Does anyone have any additional feedback they would like to share?"

- <u>Qualtrics survey</u>
    - "This next section focuses on the online Qualtrics survey. As a reminder, all Veterans who access a HIV self-test from the vending machine will have the opportunity to complete an anonymous online survey for $20. They can take the survey as many times as they would like and receive up to a maximum of $40."
    - *General experience and perceptions*
        - "How would you describe your overall experience completing this survey?
        - What were your first impressions?
        - What aspects of the survey did you find more or less relevant?"
    - *Clarity and structure*
        - "Were the questions clear and easy to understand?
        - Were there any questions that were confusing or unclear?
        - Was the survey flow and structure easy to follow?"
    - *Content and relevance*
        - "Were the survey questions relevant?
        - Were there any questions that were too intrusive?
            - Was asking HIV self-test results intrusive?
        - What questions, if any, would you suggest we remove?

        - What questions, if any, would you suggest we change the wording?
        - What questions should we add?"
    - *Ease of completion and time*
        - "How long did it take you to complete the survey?
        - What type of device did you use to complete the survey?
        - Did you experience any technical issues?
        - How easy or difficult was the survey to complete?
        - What could be done to make the survey easier and faster to complete?"
    - *Suggestions for improvement*
        - "What are some things we could do to improve the survey?
        - How could we make the survey more engaging?
        - How could we make the survey more user-friendly?"
    - "That concludes my questions about the survey. Does anyone have any additional feedback they would like to share?"

- <u>Interview guide</u>
    - "This last section focuses on the qualitative interview guide."
    - *General experience and perceptions*
        - "What were your overall impressions of the interview questions?
        - What aspects of the interview questions did you find more or less relevant?"
    - *Clarity and structure*
        - "Were the questions clear and easy to understand?
        - Were there any questions that were confusing or unclear?
        - Do the interview questions flow well?
    - *Content and relevance*
        - "Were the interview questions relevant?
        - Were there any questions that were too intrusive?
        - What questions, if any, would you suggest we remove?
        - What questions, if any, would you suggest we change the wording?
        - What questions should we add?"
    - *Suggestions for improvement*
        - "What are some things we could do to improve the interview questions?
        - How could we make the interview more engaging?
    - "That concludes my questions about the interview guide. Does anyone have any additional feedback they would like to share?"

Wrap-up

- "That concludes our focus group. Thank you again for your time and participation. Your feedback and insights are valuable to ensuring our research is relevant and informed.
- You will receive a check in the mail for you $1,000 honorarium.
- We may contact you in the future to ask about your interest in contributing to contribute to written products, such as conference abstracts, posters, and presentations, as well as written manuscripts.
- If you have any questions or concerns, you are welcome to contact me after today via phone or email.

- Thanks again!

References:

- https://www.eiu.edu/ihec/Krueger-FocusGroupInterviews.pdf
- https://irep.olemiss.edu/wp-content/uploads/sites/98/2016/05/Trinity_Duke_How_to_Conduct_a_Focus_Group.pdf

- https://tracs.unc.edu/docs/recruitment/Recruitment_Designing_Effective_Recruitment_Materials_20211006.pdf
- https://www.entropik.io/blogs/a-guide-to-asking-the-right-focus-group-questions

Appendix A. Confirmation Notice for Participation

Dear ________________,

Thank you for your interest and willingness to participate in our focus group. We would like to hear your ideas and opinions about materials we plan to use in an upcoming paid research study at UCSF and San Francisco VA Medical Center. In this study, we aim to learn about Veteran interests, experiences, and perspectives accessing and using HIV self-tests from 15 harm reduction vending machines.

The focus group will consist of a moderator, moderator assistant, and 4 Veterans living with HIV. We will require approximately 5 hours of your time, including:

- 4 hours for review of materials
- 1 hour for the focus group discussion

In addition, you will have the opportunity to participate in written products, such as conference abstracts/presentations and a manuscript publication in a peer-reviewed journal. You will be compensated with a $1,000 honorarium for your time and expertise via mailed check.

To participate, please complete the following steps:

1. Complete the W9 form to receive your payment.
    a. Page 1:
        i. Section 1: Add your personal or business information.
        ii. Section 2: Add your email only. Do not change other fields.
        iii. Section 3: Check relevant boxes if completing as a business.
        iv. Section 4: Skip.
        v. Section 5: Add your name, title, date, and sign electronically.
    b. Page 2: Skip.
    c. Page 3: Skip.
    d. Email the completed W9 form to [insert name]: [insert email]. They will work with you directly if any changes are needed.
2. Before the focus group, review the attached research study materials.
    a. Recruitment fliers
    b. Education handouts
        i. HIV 101
        ii. Rapid Oral HIV Test Fact Sheet
        iii. TakeMeHome Handout
        iv. HIV testing and treatment resources by county: Alameda, Humboldt, Lake and Mendocino, San Francisco, and Sonoma Counties
    c. Qualtrics survey
        i. Direct link: https://ucsf.co1.qualtrics.com/jfe/form/SV_0voYF7pldkmT7GS

   ii. Or via QR Code:
   iii. Electronic/paper copy is attached for reference
  d. Qualitative interview guide
3. Document your feedback on the research study materials in the attached feedback form. Email the completed form **before the focus group** to [insert name], [insert email].
4. Attend the 1-hour focus group scheduled Thursday September 18th at 5-6pm via Zoom. Be prepared to discuss what you documented in the feedback form.

If you have any questions, concerns, or are unable to attend, please contact [insert name and email]. Otherwise, we look forward to meeting with you!

Sincerely,

[insert PI name] and Research Team

Appendix B. Recruitment Tracking Form

Date	Name	Participant Number	Phone	Email	Able to Participate?			Mailing Address	Date Instructions Sent
					Yes	No	Comments		

Appendix C. Reviewer Feedback From

Product To Review	Questions for Reviewers	Reviewer Feedback<br>Instructions: Type or write your responses here.
Recruitment Fliers	• Do the fliers sufficiently answer these key questions about the research study?<br>○ What is the study about?<br>○ What do you want from me?<br>○ Why should I care?<br>○ Who is eligible to participate?<br>○ Where/how do I get involved?	
	• Are the images/pictures on the fliers relevant?<br>• Are the images/pictures on the fliers representative of:<br>○ Diverse populations<br>○ Veterans<br>○ People living with HIV	

Product To Review	Questions for Reviewers	Reviewer Feedback<br>Instructions: Type or write your responses here.
Recruitment Fliers	• Are the fliers eye catching? Do they grab your attention?<br>• Do the fliers have a good balance of images and white space?<br>• Do you like the colors and fonts used on the fliers?	
	• Are the fliers easy to read?<br>• Is the content on the fliers easy to understand?<br>• Is the language on the fliers too technical?	

Product To Review	Questions for Reviewers	Reviewer Feedback<br>Instructions: Type or write your responses here.
Recruitment Fliers	• Are the fliers easy to act on?<br>• Is having the QR code useful?<br>○ Is the website useful?<br>• Is there another/better way to share information about the study?	
	• What are some things we could do to improve the fliers?<br>• How could we make the fliers more engaging	

Product To Review	Questions for Reviewers	Reviewer Feedback<br>Instructions: Type or write your responses here.
**Education Handouts**	• What were your overall impressions of the handouts?<br>• What aspects of the handouts did you find more or less relevant?	
	• Were the handouts clear and easy to understand?<br>• Were there any handouts that were confusing or unclear?	

Product To Review	Questions for Reviewers	Reviewer Feedback<br>Instructions: Type or write your responses here.
Education Handouts	• Were the handouts relevant?<br>• Was the content in the handouts an appropriate level? Do we need to provide more educational information?<br>• What handouts, if any, would you suggest we remove?<br>• What types of handouts should we add?”	
	• What are some things we could do to improve the handouts?<br>• How could we make the handouts more engaging?	

Product To Review	Questions for Reviewers	Reviewer Feedback<br>Instructions: Type or write your responses here.
Qualtrics Survey	• How would you describe your overall experience completing this survey?<br>• What were your first impressions?<br>• What aspects of the survey did you find more or less relevant?	
	• Were the questions clear and easy to understand?<br>• Were there any questions that were confusing or unclear?<br>• Was the survey flow and structure easy to follow?	

Product To Review	Questions for Reviewers	Reviewer Feedback<br>Instructions: Type or write your responses here.
**Qualtrics Survey**	• Were the survey questions relevant?<br>• Were there any questions that were too intrusive?<br>○ Was asking HIV self-test results intrusive?<br>• What questions, if any, would you suggest we remove?<br>• What questions, if any, would you suggest we change the wording?<br>• What questions should we add?	
	• How long did it take you to complete the survey?<br>• What type of device did you use to complete the survey?<br>• Did you experience any technical issues?<br>• How easy or difficult was the survey to complete?<br>• What could be done to make the survey easier and faster to complete?	

Product To Review	Questions for Reviewers	Reviewer Feedback<br>Instructions: Type or write your responses here.
**Qualtrics Survey**	• What are some things we could do to improve the survey?<br>• How could we make the survey more engaging?<br>• How could we make the survey more user-friendly?	

Product To Review	Questions for Reviewers	Reviewer Feedback<br>Instructions: Type or write your responses here.
Interview Guide	• What were your overall impressions of the interview questions?<br>• What aspects of the interview questions did you find more or less relevant?	
	• “Were the questions clear and easy to understand?<br>• Were there any questions that were confusing or unclear?<br>• Do the interview questions flow well?	

Product To Review	Questions for Reviewers	Reviewer Feedback<br>Instructions: Type or write your responses here.
Interview Guide	• Were the interview questions relevant?<br>• Were there any questions that were too intrusive?<br>• What questions, if any, would you suggest we remove?<br>• What questions, if any, would you suggest we change the wording?<br>• What questions should we add?”	
	• What are some things we could do to improve the interview questions?<br>• How could we make the interview more engaging?	
