# Supplementary material for: Use of Vending Machines to Deliver Oral Rapid HIV Self-Tests to Veterans: Protocol for a Pilot Study
Source: JMIR Res Protoc. 2026 Jan 29;15:e84317. doi: 10.2196/84317 (PMC12855721; doi:10.2196/84317)
Supplement: Peer Review Report 1 [file resprot-v15-e84317-s002.pdf]

Applicant Full Name: Tessa Rife-Pennington
Institution: University of California, San Francisco
Application ID: H25TC8886
Application Title: CHRP 2024-25 - **Low Barrier Technology Interventions**

Percentile Rank of Average Weighted Score: 55%
Average Weighted Score: 3.15

Average Component Scores:
- Q1, **Innovation and Technology Use** (40% Weight): 3.18
- Q2, **Significance and Impact** (20% Weight): 2.82
- Q3, **Overall Research Plan** (30% Weight): 3.36
- Q4, **Management and Dissemination Plans** (10% Weight): 3.09

Technical Summary (Provided by Applicant)

In 2022, California had the highest prevalence of people living human immunodeficiency virus (HIV) of all states. There were 4,882 new HIV diagnoses, and rates were highest in cisgender men, Black/African American and Latinx individuals, and men who have sex with men (MSM). HIV prevalence is also high among Veterans with history of opioid/alcohol use disorders, hepatitis B/C virus and sexually transmitted infections, and homelessness. HIV testing is essential for early detection and to reduce morbidity, mortality, and costs. Yet in 2020, approximately 12% of people living in California with HIV were unaware of their infection. As of 2023, 50% of Veterans in care had never been tested for HIV.

Barriers to HIV testing include stigma, racism, homophobia, and lack of cultural competence in healthcare settings; lack of knowledge or access; cost and confidentiality concerns; low income, education, or perceived benefit; unemployment; and incarceration. Despite barriers, Veterans often want HIV testing and to know their results. Use of HIV self-tests (HIVST), where a person self-collects an oral fluid sample, tests, and reads the results, is an evidence-based strategy to increase testing uptake and address barriers. Further, distribution via vending machines (VMs) is an innovative, low barrier, technology-based approach demonstrated to increase confidentiality, privacy, and convenience. It is feasible, acceptable, and preferable over in-clinic testing, which facilitates engagement with first-time testers and hard-to-reach individuals, and those with a positive test often seek further testing/treatment.

Most evidence for VM-dispensed HIVSTs is among MSM, in the United Kingdom, Africa, and Los Angeles, California, and in commercial sex venues/public spaces. No studies have examined impacts of Veteran self-administered HIV tests or distribution via VMs. We aim to bridge this gap by evaluating the first VM-dispensed HIVSTs designed for Veterans, located in Northern California, and in healthcare and supportive housing settings. We will collect Veteran self-report data via electronic surveys and HIVST distribution data via software from 15 VMs to evaluate Reach, Effectiveness, Adoption, Implementation, and

Maintenance (RE-AIM). Study results may inform expansion of HIVST availability and delivery via VMs for Veterans and in healthcare and community-based settings.

Critiques: Primary Reviewer

Critique Text: This study focuses on adding HIV self-test kits to harm reduction vending machines that are being utilized in health care settings and supportive housing settings that are used by veterans and collecting data on utilization. The idea of including HIV self-test kits in vending machines that are used by veterans is novel and has the potential for high impact. Veterans are an important population who could benefit from the addition of safe sex kits in the vending machines. The team is strong and has expertise working with the target population. Enthusiasm is dampened by insufficient information regarding the applicant's justification that the desired sample size would be achieved and the focus on only 90 veterans for the analysis. The addition of the veterans that would be used as advisors is strong, but more attention to their specific role in the project would have enhanced the application.

Critique Questions:

Q1: Please score and comment on Component 1 - Innovation and Technology Use: Degree of innovation in the proposed work, the appropriateness of the technology to be deployed, and the degree of innovation that incorporation of adding this technology to service delivery would bring to the field.
Weight: 40%
Answer: The concept of the study, adding HIV self-test kits to existing harm reduction vending machines is novel and it has the potential to improve access to HIV testing. Use of existing vending machines takes advantage of existing resources. The applicant did not adequately address the potential technological barriers to using the vending machines (i.e. vandalism, maintenance in rural settings) nor did they address any support mechanism that could be integrated into the machines to provide the users with information on additional resources or HIV prevention services.

Q2: Please score and comment on Component 2 - Significance and Impact: Significance of the problem (e.g. the barrier to care) that would be addressed, the degree to which the study population is significant in the California HIV epidemic, the potential impact of the technology-based solution to decreasing barriers in service delivery to the priority population, the representativeness of the research team, and the extent to which the research team includes meaningful engagement with community partners.
Weight: 20%
Answer: Veterans are an important priority populations that is needed of HIV and harm reduction resources. The applicant provided evidence that the harm reduction vending machines have been useful in distributing harm reduction equipment and it stands to reason that the addition safe sex kits in these machines could also be useful. The main reservation is that the applicant did not adequately describe how adding the self-testing kits would result in the desired effect. The applicant did not discuss any strategies for heightening awareness of the kits. With some marketing and information dissemination adding the self-testing kits to the machines may be a useful strategy for increasing

testing. However, more is needed to ensure that veterans know about these kits. The technology is scalable and if the results show promise it could be disseminated widely. The research team is diverse and appropriate. The applicant stated in the budget that they will engage 4 veteran advocates but did not describe their role in the project.

Q3: Please score and comment on Component 3 - Approach and Feasibility/Overall Research Plan: Appropriateness and scientific rigor of the overall approach(es) used to address stated aims; and the feasibility and rigor proposed to execute all aspects of the investigation. For projects proposing scale up or expansion of existing programs, the research plan should also include plans for sustainability. This section should provide descriptions and justifications for sampling and recruitment strategies, statistical approaches, and project timelines.
Weight: 30%
Answer: The applicant provided strong preliminary evidence for the approach described in the application. The vending machines have been useful for distributing harm reduction equipment. The application could have been strengthened by including more details on the procedures being used to let the veterans know that the kits were added to the vending machines. They also did not provide a strong rationale for its exclusive focus on HIV self-testing without addressing additional services such as PrEP. The applicant states that they will distribute 900 HIV prevention kits and that 90 veterans would complete the survey but provided insufficient evidence to support these numbers.

Q4: Please score and comment on Component 4 - Approach and Feasibility/Management and Dissemination Plans: The capacity of the team to carry out the work; the environment in which the work will be done; how the project will be managed; and strategies for addressing roadblocks that may impact completion of aims. Details for sharing the results of the work to all relevant stakeholders in the dissemination plan.
Weight: 10%
Answer: The PI is a new investigator who is supported by an established group of mentors. The applicant provides insufficient specificity regarding project management outside of meeting nor did the applicant address roadblocks that may impact completing the aims. The applicant included minimal details regarding the dissemination plan and the plan lacked a timeline.

Unscored Mandatory Questions:

Q1: Please comment on: • Safety and Inclusion
Answer: No concerns

Q2: Please comment on: • Budget
Answer: No concerns

Q3: Please comment on: • Scientific or Budgetary Overlap
Answer: No concerns

Q4: Please comment on: • Research Conducted Outside of California
Answer: No concerns

Critiques: Secondary Reviewer

Critique Text: This proposal, led by Dr. Tessa Rife-Pennington from UCSF, outlines a well-structured and innovative plan to evaluate the feasibility, reach, and adoption of HIV self-tests (HIVSTs) distributed through Harm Reduction Vending Machines (HRVMs) among Veterans in Northern California. The project addresses the disproportionately high rates of HIV-related risk factors among Veterans and aligns with the Ending the HIV Epidemic (EHE) initiative. By leveraging low-barrier technology, the project seeks to increase HIV testing access for Veterans in underserved and rural areas. The use of the RE-AIM framework adds rigor to the evaluation, ensuring that the intervention's long-term feasibility and sustainability are assessed.

Strengths:
1. Innovation and Technology Use: The project employs an innovative use of HRVMs to distribute HIVSTs, allowing for anonymous, 24/7 access to testing. This technology is particularly suited to Veterans who face barriers such as stigma, transportation challenges, and limited healthcare hours. The inclusion of the RE-AIM framework ensures a structured evaluation of the intervention, and the scalability of the HRVMs increases the potential for long-term impact.
2. Significance and Impact: The project addresses a critical public health need by targeting Veterans, a population with high rates of HIV-related risk factors. The focus on underserved and rural Veterans increases the equity impact of the intervention. The project is aligned with both U.S. and California's EHE initiative, contributing to early diagnosis and prevention efforts.
3. Approach and Feasibility: The proposal presents a well-defined research plan, with a clear recruitment strategy and strong institutional support from the SFVAHCS. The PI's prior experience with harm reduction initiatives adds to the project's feasibility, and the inclusion of Veteran Advocates ensures the intervention is responsive to the needs of the target population.
4. Management and Dissemination Plans: The project includes a solid management plan, with regular team meetings and dedicated staff for HRVM maintenance. The dissemination plan is well-rounded, targeting academic, community, and Veteran audiences. The use of participatory action research (PAR) and Veteran Advocates enhances community engagement and ensures findings are shared in an accessible manner.

Weaknesses:
1. Cultural and Population Diversity: While the project focuses on Veterans as a group, it does not sufficiently address the diversity within this population, such as women, LGBTQIA+ Veterans, or racially and ethnically diverse Veterans. Tailoring the intervention to ensure representation from diverse subgroups would improve the inclusiveness of the study.
2. Linkages to Care and Policy Impact: The proposal could benefit from a more detailed plan for linking Veterans who test positive to follow-up care, including HIV treatment and Pre-Exposure Prophylaxis (PrEP). Additionally, there is limited discussion of how the findings will influence broader VA or national policy changes, which could enhance the long-term impact of the intervention.
3. Short-Term Focus on Feasibility: While the project lays a solid foundation for future expansion, its

immediate impact is limited to assessing feasibility and adoption. Broader public health outcomes, such as reductions in HIV transmission, will not be measured within this study phase.

This proposal demonstrates an innovative approach with significant potential to improve HIV prevention efforts among Veterans. While there are minor weaknesses related to diversity inclusion, care linkages, and policy engagement, the project is feasible, well-structured, and aligned with public health priorities. With future iterations, the project has strong potential for scalability and broader public health impact.

Critique Questions:

Q1: Please score and comment on Component 1 - Innovation and Technology Use: Degree of innovation in the proposed work, the appropriateness of the technology to be deployed, and the degree of innovation that incorporation of adding this technology to service delivery would bring to the field.
Weight: 40%
Answer: • Innovative Use of Vending Machines for HIVST: The proposal uses Harm Reduction Vending Machines (HRVMs) to dispense HIV self-tests (HIVSTs), which is an innovative, low-barrier, and confidential approach. The vending machines allow for 24/7 access, which overcomes many barriers faced by Veterans, such as stigma, lack of transportation, and limited healthcare hours. The use of HRVMs in this context is novel, particularly for targeting underserved Veterans in both urban and rural areas. • Tailored for Veterans: This innovation is tailored specifically to Veterans who may face additional barriers to healthcare, such as mental health issues, housing insecurity, or stigmatization from past drug use or sexual behavior. By focusing on this population, the technology aligns closely with the unique challenges Veterans experience in accessing preventive care. • Evidence-Based: The project builds on existing evidence that demonstrates the feasibility, acceptability, and efficacy of vending machine-dispensed HIVSTs in other settings. Studies from the U.K., Los Angeles, and Africa support the potential for vending machines to increase testing uptake, particularly among marginalized populations such as men who have sex with men (MSM) and people who use drugs (PWUD). Applying this technology in the Veteran population is a logical and innovative extension. • Integration of RE-AIM Framework: The use of the RE-AIM framework is a strength in that it adds rigor to the evaluation of this innovative technology. This systematic approach will evaluate the reach, adoption, and sustainability of HRVMs, ensuring the intervention's long-term viability and impact. • Low-Cost, Sustainable Model: The technology is positioned as a cost-effective and scalable solution. The vending machines can be stocked and maintained at a low cost, with minimal human intervention, making this an attractive option for long-term, sustainable implementation in both healthcare and supportive housing settings.
Weaknesses: • Limited Discussion of Potential Technological Barriers: While the vending machine approach is promising, the proposal does not thoroughly address potential technological challenges, such as vandalism, malfunction, or the need for ongoing maintenance in remote or rural areas. These logistical challenges could impact the sustained use and adoption of the technology, particularly in underserved areas with less infrastructure support. • No Discussion of App or Digital Support: The proposal could be strengthened by including a discussion of potential digital (or non-digital) support mechanisms, such as an app or web portal, that could integrate with the HRVM system. Offering a digital platform where Veterans can access additional resources (e.g., mental health services, HIV

prevention education) or track their use of the vending machines might enhance user engagement and support adherence to HIV prevention measures. This proposal demonstrates a highly innovative approach to HIV prevention using low-barrier technology. The tailored focus on Veterans and the integration of the RE-AIM framework strengthens the project's design. However, addressing potential logistical barriers and considering the integration of digital tools could enhance the project's long-term success and scalability.

Q2: Please score and comment on Component 2 - Significance and Impact: Significance of the problem (e.g. the barrier to care) that would be addressed, the degree to which the study population is significant in the California HIV epidemic, the potential impact of the technology-based solution to decreasing barriers in service delivery to the priority population, the representativeness of the research team, and the extent to which the research team includes meaningful engagement with community partners.
Weight: 20%
Answer: • Addresses Critical Public Health Need: The project addresses a significant public health concern by focusing on Veterans, a population with disproportionately high rates of HIV-related risk factors, including substance use disorders, mental health issues, and homelessness. The proposal highlights the low rates of HIV testing among Veterans, with only 50% having ever been tested, despite VA recommendations for regular testing. By increasing access to HIV self-testing, the project has the potential to reduce HIV transmission and improve early detection. • Supports the Ending the HIV Epidemic (EHE) Initiative: The intervention directly aligns with both the U.S. and California's Ending the HIV Epidemic (EHE) initiative. By offering a novel approach to expanding HIV testing access through HRVMs, the project addresses one of the key pillars of the EHE—early diagnosis. This strengthens the significance of the project in the broader context of national and state-level HIV prevention efforts. • Reaches Marginalized and High-Risk Populations: Veterans, especially those in supportive housing and rural areas, often face multiple barriers to accessing healthcare, including stigma, transportation challenges, and distrust of traditional healthcare systems. This project provides a low-barrier, confidential way for these individuals to access critical HIV prevention tools, making it particularly impactful for marginalized populations. The focus on underserved, rural, and marginalized Veterans increases the equity impact of the project. • Scalability and Broader Impact Potential: The project is designed to be scalable. If successful, HRVMs could be implemented across other VA facilities and community-based programs. The potential for broader adoption across other high-risk populations and geographic areas adds to the project's long-term public health impact. Weaknesses: • Short-Term Focus on Feasibility: While the project addresses an important issue, its immediate impact is somewhat limited by its focus on feasibility and adoption. Although necessary as a foundational step, broader outcomes, such as reductions in HIV transmission or improvements in long-term care engagement, will not be measured in this study. This limits the immediate public health significance of the current phase of the project. • Limited Engagement with HIV Prevention Beyond Testing: While the focus on HIV self-testing is critical, the proposal does not provide substantial details on how the project will link Veterans to preventive services such as Pre-Exposure Prophylaxis (PrEP) or post-test care, particularly for those who test positive. Including stronger mechanisms for linking Veterans to comprehensive HIV prevention and care services would improve the long-term impact of the intervention. The project addresses a critical public health need and has strong alignment with national and state-level HIV prevention goals.

Its focus on reaching high-risk and underserved populations enhances its significance. However, the immediate focus on feasibility limits short-term impact, and greater attention to linking Veterans to comprehensive HIV care would improve the project's overall reach.

Q3: Please score and comment on Component 3 - Approach and Feasibility/Overall Research Plan: Appropriateness and scientific rigor of the overall approach(es) used to address stated aims; and the feasibility and rigor proposed to execute all aspects of the investigation. For projects proposing scale up or expansion of existing programs, the research plan should also include plans for sustainability. This section should provide descriptions and justifications for sampling and recruitment strategies, statistical approaches, and project timelines.
Weight: 30%
Answer: • Well-Defined Research Plan: The proposal provides a detailed and well-organized plan to evaluate the feasibility, reach, and adoption of HRVMs through a descriptive, observational, prospective cohort study. The use of the RE-AIM framework (Reach, Effectiveness, Adoption, Implementation, and Maintenance) ensures a structured and systematic evaluation of both individual and organizational factors that influence the success of the intervention. • Strong Recruitment Strategy: The recruitment plan is practical and achievable, with an existing base of nearly 400 Veterans already registered for HRVM access, and the expectation of 700-800 by the start of data collection. This ensures the project has a solid foundation for collecting robust data. The proposal outlines the use of outreach through VA and community organizations, which should facilitate successful recruitment, particularly in rural and supportive housing settings. • Experienced PI and Team: The principal investigator (PI), Dr. Tessa Rife-Pennington, has demonstrated experience in harm reduction interventions and has already piloted successful harm reduction strategies (e.g., naloxone distribution, fentanyl test strips) for Veterans in collaboration with VA leadership. The team also benefits from the mentorship of an experienced clinician with over 20 years of HIV patient care and implementation science experience, which adds to the project's feasibility. • Veteran Advocate Engagement: The inclusion of Veteran Advocates with lived experience of HIV is a strength of the approach. These advocates will provide feedback on survey design, educational materials, and help with dissemination efforts. This community-engaged approach ensures that the intervention is responsive to the needs of the target population and is likely to increase the intervention's acceptability among Veterans. • Practical Use of Technology: The proposal demonstrates the feasibility of adapting the existing HRVMs to stock HIVSTs. The PI has prior experience in modifying and managing these machines, which reduces the risk of logistical issues. The technology has already been successfully piloted for other harm reduction supplies, indicating that the expansion to HIVSTs is a logical and achievable step. Weaknesses: • Limited Discussion of Veteran Subpopulations: While the project focuses on Veterans as a population, there is limited discussion of how the study will account for the diversity within this group. Veterans are not a monolith, and the proposal does not address how it will ensure representation from various subpopulations, such as women, LGBTQIA+ Veterans, or racially and ethnically diverse Veterans. Tailoring the intervention and outreach to ensure inclusion of diverse groups could strengthen the approach. • No Quantitative Measures of Cost-Effectiveness: While the proposal discusses cost savings, it could benefit from including quantitative measures of cost-effectiveness as part of the evaluation. This would provide valuable insights into the economic sustainability of the HRVM intervention, particularly for scaling the program to other VA facilities or healthcare settings. • Opportunities for Strengthening Linkages to Care: Although the study

measures focus on uptake and use of HIVSTs, there is little detail on how Veterans who test positive will be linked to follow-up care, such as HIV treatment or PrEP. A more explicit plan for integrating the intervention with existing VA or community-based services would improve the feasibility of ensuring that those who test positive receive the necessary care. The approach is comprehensive and feasible, with strong leadership and a clear recruitment plan. However, the proposal could benefit from more explicit attention to the diversity of Veterans and a more detailed plan for linking Veterans to follow-up HIV care. Including quantitative cost-effectiveness analysis would further strengthen the approach.

Q4: Please score and comment on Component 4 - Approach and Feasibility/Management and Dissemination Plans: The capacity of the team to carry out the work; the environment in which the work will be done; how the project will be managed; and strategies for addressing roadblocks that may impact completion of aims. Details for sharing the results of the work to all relevant stakeholders in the dissemination plan.

Weight: 10%

Answer: • Experienced Institutional Support: The project is based at the San Francisco Veterans Affairs Health Care System (SFVAHCS), a well-established institution with a strong infrastructure for supporting research, particularly in HIV prevention and harm reduction. The proposal highlights the SFVAHCS's capacity to manage the project, including the existing infrastructure for HIV care and the successful implementation of prior harm reduction initiatives led by the PI. • Clear Management Plan: The management plan is well-structured, with regular meetings between the PI, mentor, and team members to monitor progress and address potential issues. The inclusion of dedicated staff for HRVM maintenance and monitoring ensures that logistical challenges (e.g., restocking, software updates) are addressed in a timely manner. The PI's previous experience with managing HRVMs reduces the risk of delays or operational issues. • Comprehensive Dissemination Strategy: The proposal includes a thoughtful dissemination plan that targets a broad range of audiences, including academic researchers, Veterans, and community stakeholders. The plan includes presentations at scientific conferences (e.g., the American Conference for the Treatment of HIV) and publications in peer-reviewed journals (e.g., the Journal of the International AIDS Society). Additionally, the inclusion of Veteran Advocates in the dissemination process ensures that the findings are shared with the Veteran community in an accessible and meaningful way. • Community Engagement: The use of participatory action research (PAR) methodology, with Veteran Advocates providing feedback and support, is a strength of the dissemination plan. Engaging Veterans with lived experience in HIV care ensures that the materials and findings are aligned with the needs of the target population, increasing the likelihood of community buy-in and adoption. Weaknesses: • No Detailed Timeline for Dissemination: While the dissemination plan outlines several key activities (e.g., conference presentations, journal publications), it would benefit from a more detailed timeline or specific milestones for when these activities will occur. This would provide greater clarity on the project's dissemination process and ensure that findings are shared in a timely manner. (minor feedback) • Limited Focus on Policy Impact: The proposal does not provide a strong focus on how the findings will inform broader policy changes within the VA or at the state/national level. While the results will be shared with internal VA audiences, a more explicit plan for how the project might influence VA policies on HIV prevention or harm reduction could enhance its long-term impact. The management and dissemination plans are strong, with clear leadership and a

solid dissemination strategy. The use of Veteran Advocates is a key strength, but the plan could benefit from a more detailed dissemination timeline and a stronger focus on policy impact

Unscored Mandatory Questions:

Q1: Please comment on: • Safety and Inclusion
Answer: no concerns

Q2: Please comment on: • Budget
Answer: no concerns

Q3: Please comment on: • Scientific or Budgetary Overlap
Answer: no concerns

Q4: Please comment on: • Research Conducted Outside of California
Answer: no concerns

Critiques: Tertiary Reviewer

Critique Text: No Comment

Critique Questions:

Q1: Please score and comment on Component 1 - Innovation and Technology Use: Degree of innovation in the proposed work, the appropriateness of the technology to be deployed, and the degree of innovation that incorporation of adding this technology to service delivery would bring to the field.
Weight: 40%
Answer: This proposal fits under 'innovative use of technology to delivery existing prevention/care tools.' The key innovation for this study is analyzing vending machine-dispensed HIVSTs (self-tests) among Veterans. The gap in knowledge is that Veterans face additional barriers to HIV testing, with only 50% of Veterans having ever been tested for HIV. It is unknown how VM-dispensed HIVSTs among Veterans in Northern California might improve testing accessibility and knowledge of HIV status. There are 15 existing VMs in the target area, and this research can inform the expansion and use of HIVST for Veterans in other healthcare or community settings. Although VMs in harm reduction are an existing intervention, they are still novel, and much of their impact on HIV morbidity is still unknown, for both short-term and long-term outcomes. Veterans are a unique population of persons living with HIV that are further difficult to study because of VA versus non-VA care delineation, often prohibiting research on clinical indicators or widespread public health initiatives that service both populations. This proposal would bridge these gaps through primary data collection in an intervention that is accessible to Veterans in relatively high-risk populations (e.g., intermittent housing, rural). The use of RE-AIM framework to analyze the intervention, along with validated questionnaires, strengthens the

effectiveness of program evaluation.

Q2: Please score and comment on Component 2 - Significance and Impact: Significance of the problem (e.g. the barrier to care) that would be addressed, the degree to which the study population is significant in the California HIV epidemic, the potential impact of the technology-based solution to decreasing barriers in service delivery to the priority population, the representativeness of the research team, and the extent to which the research team includes meaningful engagement with community partners.
Weight: 20%
Answer: Preliminary groundwork by the investigators suggests Veterans demonstrate high acceptance of HIV rapid oral testing, and in-home, self-administered HIV tests demonstrate high specificity and sensitivity. This foundation also demonstrates knowledge and prior experience working with their target population to build rapport and trust. Earlier identification of HIV status, particularly in Veterans facing unique comorbidities from military culture and/or experiences of war trauma, can improve not only 'known status' but the ability of public health outreach to link them to care. VMs are a unique opportunity to distribute these test kits to Veterans because they help overcome mistrust of healthcare delivery systems or might be the only point of contact for preventive services for Veterans outside of the medical care systems. They will recruit Veteran advocates with lived experience with HIV and include educational hands about follow-up treatment that will be culturally sensitive to the needs of this population while advocating for key services (ART, linkage to care, PrEP), a highlighted strength to the rollout and feasibility of this project through peer consultation of the target population. A cost analysis demonstrates estimated savings of ~$100,000 in STI-attributable HIV costs based on the number of Veterans served by the SFVAHCS and preliminary counts (STI-attributable HIV infections). This proposal also emphasizes the accessibility to marginalized Veterans, meaning Veterans of any healthcare eligibility status can register/access the HRVMs, thereby increasing testing among these groups. They also highlight the rural accessibility of some HRVMs. It is of note that, although data measures about the testing results and the participant's plan of action in the event of a positive are being collected through the survey, there is a gap in being able to measure linkage to care or whether appropriate public health follow up is initiated.

Q3: Please score and comment on Component 3 - Approach and Feasibility/Overall Research Plan: Appropriateness and scientific rigor of the overall approach(es) used to address stated aims; and the feasibility and rigor proposed to execute all aspects of the investigation. For projects proposing scale up or expansion of existing programs, the research plan should also include plans for sustainability. This section should provide descriptions and justifications for sampling and recruitment strategies, statistical approaches, and project timelines.
Weight: 30%
Answer: The researchers bring a wealth of experience in working with Veterans and Veteran providers, conducting focus groups, establishing feedback loops, developing HRVMs (harm reduction vending machines), and conducting outreach with special populations (Veterans, unhoused Veterans, substance-using Veterans, etc.). The supplies to be included in these HRVMs were also selected by Veteran feedback surveys, demonstrating previous sampling and recruitment success. There is strong engagement with the Veteran population who might be at risk for HIV (regardless of VA enrollment), a

difficult to reach subgroup, and the researchers demonstrate the ability to incorporate this feedback and speak to the needs of the Veterans. Previous studies have used electronic surveys to collect Veteran registration, with about 281 Veterans using machine access in about an 8-month period (prior study), with most access in non-medical locations. The sample size is justified (900 HIVSTs distributed, 90 electronic surveys completed) based on this previous groundwork and recognizing that each Veteran can receive up to one HIVST test per week. The use of the RE-AIM framework has the capacity to appropriately measure the impacts of the program, and outcome measures are clearly defined. The statistical approach is weaker, with only descriptive measures being used to determine feedback. It is arguable that this first step will lead to more robust study design for future expansion, but that is not detailed. There appear to be some missing indicators, such as information on military service-related indicators. Literature on the age, deployment, rank, etc., influence the Veteran experience and, subsequently, health and living experiences of this group.

Q4: Please score and comment on Component 4 - Approach and Feasibility/Management and Dissemination Plans: The capacity of the team to carry out the work; the environment in which the work will be done; how the project will be managed; and strategies for addressing roadblocks that may impact completion of aims. Details for sharing the results of the work to all relevant stakeholders in the dissemination plan.
Weight: 10%
Answer: There is experience in not only designing the distribution of HIVST kits through the VM but stakeholder engagement to determine their locality and distribution. The software is set up to collect data anonymously, with previous work in establishing the data collection methods (REDCap, VendNovation web-based software). The data collection is in line with the distribution of supplies being free of cost and anonymous. Dissemination includes Veteran advocate. The dissemination plan lacks detailed description, but staff has experience in implementation and dissemination science. The research team comprises of local experts as well as VA-wide staff to guide the principles of the work. Part of the plan for rural access via the location of the HIVST at HRVMs in non-urban or suburban counties also links to some lived experiences of the investigators (WVU).

Unscored Mandatory Questions:

Q1: Please comment on: • Safety and Inclusion
Answer: The inclusion of Veterans is robust, touching upon groups that may or may not be connected to health care or medical services. The security and confidentiality is the largest risk to safety, but data will be stored in a protected file on the SFVAHCS network with restricted access to study staff. Identifying information will only be used to assign alpha-numeric identifiers and destroyed upon completion. The dispensing data will be anonymous.

Q2: Please comment on: • Budget
Answer: The budget plan includes required personnel as well as advocate costs and supply costs, gift card compensation.

Q3: Please comment on: • Scientific or Budgetary Overlap
Answer: No overlap identified

Q4: Please comment on: • Research Conducted Outside of California
Answer: The research is conducted in California with some mentorship from VA-personnel (no costs) in locations external to California.

Critiques: Community Reviewer

Critique Text: Proposal strengths: 1) Focuses on the Veteran population, a population that has limited data regarding specific HIV and Harm reduction needs, 2) Will target the rural, suburban and urban areas in North California 3) Targets areas/locations where Veterans are provided housing/housing resources 4) Proposes to reduce barriers to accessing HIV related care/services/resources for Veterans 5) Proposes to improve immediate and free access to HIVST kits and better access to care/treatment if needed 6) PrEP info will be provided too 7) Incentives provided to participants and honorariums provided to participating Veterans living with HIV 8)Peer engagement/Veteran feedback of pros and areas of improvement

Concern: I see the research demographics reflecting 300+ people but the project sample/surveying sample is 90 people (10% of the 900 surveys). Does the research team know if the sample size will reflect that women and minorities are captured?

Critique Questions:

Q1: Please score and comment on Component 1 - Innovation and Technology Use: Degree of innovation in the proposed work, the appropriateness of the technology to be deployed, and the degree of innovation that incorporation of adding this technology to service delivery would bring to the field.
Weight: 40%
Answer: Awesome idea to focus specifically on Veterans and their HIV/Harm reduction related needs, especially take home/self-administering HIV testing services! Good to see that this project will focus on serving Veterans in the suburban, urban and rural areas in North California. To collaborate with other service and housing providers to utilize and maintain HIV/Harm reduction Vending machines (HRVMs) already established is an innovative low-barrier intervention idea. Good to see that these vending services will be free. Using Veterans that are living with HIV to help with project feedback, improvements and engaging other Veterans is a productive strategy!

Q2: Please score and comment on Component 2 - Significance and Impact: Significance of the problem (e.g. the barrier to care) that would be addressed, the degree to which the study population is significant in the California HIV epidemic, the potential impact of the technology-based solution to decreasing barriers in service delivery to the priority population, the representativeness of the research team, and the extent to which the research team includes meaningful engagement with community partners.

Weight: 20%
Answer: The research team has extensive contributions to science and experience in HIV and Harm reduction related services and research. The details of their previous harm reduction research work are beneficial to help carry out the proposed plan. Support letters are included that are from community partners that will help/mentor the research team carry out proposed activities. The research team has noted how Veterans in California have faced barriers to care including, dealing with addictions, racism, not being offered HIV testing while in care, limited to none research about Veterans and HIV, and fear of HIV test results and potential consequences, just to name a few. The plan also notes that "HIV prevalence is high among Veterans with history of opioid and/or alcohol use disorders, hepatitis B or C virus (HBV, HCV) infection, sexually transmitted infections (STIs), pneumonia, and homelessness." The proposed plan wants to address these barriers with the implementation of Self-administered/take home HIV Test kits in HRVMs, free to the populations served. Veterans can learn their HIV status and seek care and treatment when using these self-testing kits. Providing access to these kits are also a strategy to help reduce HIV transmission and increase access to care/treatment. The locations the HRVMS are placed in locations that would increase access to these kits and resources. Other strategies/tools that this plan proposes to utilize to address the barriers: surveys, community partnerships and engagement from peers that are Veterans. The proposed info/resource pamphlet that's attached to the testing kit should be a neon color and has an attracting statement like 'earn a $$ gift card' in big and bold letters on the front, so that the person can actually open and read it instead of tossing it because they don't feel like reading 'more stuff'. It's also possible that some Veterans may not be able to read or understand what the pamphlet is asking them to do, will this pamphlet be simple to read? Will some of veterans be tech savvy to follow internet related instructions?

Q3: Please score and comment on Component 3 - Approach and Feasibility/Overall Research Plan: Appropriateness and scientific rigor of the overall approach(es) used to address stated aims; and the feasibility and rigor proposed to execute all aspects of the investigation. For projects proposing scale up or expansion of existing programs, the research plan should also include plans for sustainability. This section should provide descriptions and justifications for sampling and recruitment strategies, statistical approaches, and project timelines.
Weight: 30%
Answer: The researcher presented data that has shown the need for the proposed activities, strategies and resources to support the idea of the HRVM low barrier tech intervention for California veterans. The timeline and proposed plan include information: appropriate and viable locations of HRVMs in Veteran centered living areas, survey tools and methods to collect data of Veterans' needs and sexual/drug behaviors, on-site recruitment of potential participants, use of Veteran peers for project input and more, maintenance of HRVM HIVst kits/supplies, dissemination of study's findings, and collaborative partnerships/mentoring to carry out the project. The researcher states that the study's results may "influence and advance traditional Harm Reduction Program service delivery within the VA and in community-based programs. In addition, the PI can utilize results generated to apply for larger-scale grants with similar aims and protocols."

Q4: Please score and comment on Component 4 - Approach and Feasibility/Management and Dissemination Plans: The capacity of the team to carry out the work; the environment in which the

work will be done; how the project will be managed; and strategies for addressing roadblocks that may impact completion of aims. Details for sharing the results of the work to all relevant stakeholders in the dissemination plan.
Weight: 10%
Answer: The well-experienced team appears to have the capacity and partnerships in place to carry this proposed project forward. The housing locations and type of housing (veterans living-centered) to reach the Veteran population seem to be ideal and appropriate for this study. Not much details about strategies to address potential limitations or obstacles beyond obtaining feedback from Veteran participants/Veteran living with HIV. Project's results will be disseminated to "internal VA audiences, to HUD-VASH housing staff/Veteran residents, at scientific conference(s) (e.g., American Conference for the Treatment of HIV, STI Engage, US Conference on HIV/AIDS), and in peer-reviewed journal(s) (e.g., Journal of the International AIDS Society, Sexually Transmitted Infections, Harm Reduction Journal)."

Unscored Mandatory Questions:

Q1: Please comment on: • Safety and Inclusion
Answer: The demographics representation of women and minorities are included but may not be completely represented in research outcomes based on how study sample may be selected. Steps to protect project participants' identities are put in place.

Q2: Please comment on: • Budget
Answer: Budget appears feasible and reasonable, well justified.

Q3: Please comment on: • Scientific or Budgetary Overlap
Answer: no comment

Q4: Please comment on: • Research Conducted Outside of California
Answer: no comment

Remarks from Program

CHRP was pleased to receive this proposal from an investigator with a great deal of clinical experience working with veterans who use drugs. Substance use is an important syndemic factor for HIV and increasing the accessibility of HIV self-test kits is both timely and relevant. Paid inclusion of veterans with lived HIV experience in both the planning and dissemination is an appreciated element of this proposal. As part of this feasibility test, it will be important to document efforts to grow the demand for HIV test kits. We would also recommend developing a plan for referral to services (PrEP or HIV treatment) following testing. In addition, reviewers recommended collecting key service indicators (branch, where and how they served, rank, deployment), which can be critically important to veteran population.

Some clarification around the sample size is needed as the sample size in the narrative (n=90) does not match the sample size in the table (n=343).
